# A Pedagogy of Preparation: Helping Underprepared Students Succeed in College-Level Coursework in Community Colleges

**DOI:** 10.1007/s10755-020-09531-9

**Published:** 2021-01-07

**Authors:** Rebecca L. Brower, Amanda N. Nix, Hollie Daniels, Xinye Hu, Tamara Bertrand Jones, Shouping Hu

**Affiliations:** Center for Postsecondary Success, Florida State University, Tallahassee, FL 32306-4452 USA

**Keywords:** Community colleges, Developmental education, Pedagogy, Instruction, Scaffolding, Collaboration

## Abstract

This paper presents an overall educational philosophy of working with students underprepared for college-level work, which we term “a pedagogy of preparation.” We consider how instructors scaffolded instruction to foster college readiness in students who were now able to enroll in college-level work regardless of academic preparation after state-level legislation (SB 1720) that dramatically altered the delivery of developmental education in the Florida College System (FCS). We also consider how collaboration increased among campus personnel after the legislation to foster college readiness in students underprepared for college-level work.

Developmental education (DE) is a postsecondary practice designed to help college students shore up academic skills so that they can successfully participate in college-level coursework. However, the effectiveness of DE has long been in question. Bettinger and Long (2005) found that enrollment in DE increases the likelihood of dropout. Furthermore, enrollment in DE has been shown to decrease the likelihood that community college students will transfer to a four-year institution and earn a four-year degree (Attewell, Lavin, Domina, & Levey, 2006; Crisp & Delgado, 2014; Melguizo, Bos, & Prather, 2011). These findings suggest that enrollment in DE may disadvantage Black, Latinx, and economically disadvantaged community college students’ long term postsecondary educational attainment considering their over-representation in DE programs (Attewell et al., 2006; Bettinger & Long, 2005; Grimes & David, 1999). Moreover, annual estimates of the costs of DE in the US range from $1.13 billion (Pretlow & Wathington, 2012) to $6.7 billion (Scott-Clayton, Crosta, & Belfield, 2014), making it a costly endeavor.

In response to these critiques, policymakers have called for reform (Bailey, Jeong, & Cho, 2010). The most recent wave of DE reform, which began nearly a decade ago, has affected community college systems nation-wide, in states such as California, Florida, North Carolina, Texas, Tennessee, and Virginia (Jaggars & Bickerstaff, 2018). Supporters of the reform have identified prolonged course sequences, flawed placement tests, and poor instruction as the primary shortcomings of DE (Bailey, 2009; Jaggars & Bickerstaff, 2018). Consequently, the most recent wave of DE reform has focused on, “revised assessment for course placement, acceleration struggles, and changes to the content and pedagogy of developmental curricula” (Jaggars & Bickerstaff, 2018, p. 478). While changes vary by state, DE reform generally seeks to revise DE curriculum and/or allow students to bypass it entirely (Bailey, 2009; Western Interstate Commission for Higher Education, 2016).

The Florida legislature passed a sweeping version of DE reform in 2013, namely Senate Bill (SB 1720), which constitutes the context for this qualitative study. Florida’s community colleges have one of the most demographically diverse student populations in the nation, reflecting the changing demographics of the rest of the United States. Thus, the findings in this study have implications not just for Florida, but for community colleges nationwide.

Even though DE policies and procedures changed following SB 1720, incoming students—and their levels of academic preparation—did not. Many still needed help getting ready for college-level coursework, even if they did not choose to enroll in DE classes. The purpose of this paper is to identify the educational philosophy that undergirded the principles and practice of how Florida colleges prepared students for college-level coursework outside of DE course pathways following SB 1720. Specifically, we seek to identify a pedagogy of preparation and consider how these practices can be infused throughout college-level curriculum and in other functional areas, including advising and academic support. In so doing, we pose three research questions:

1. What is the educational philosophy undergirding instruction for underprepared students in the Florida College System (FCS)?
2. How did FCS instructors scaffold instruction to foster college readiness in students underprepared for college-level work?
3. How did all FCS campus personnel foster college readiness in students underprepared for college-level work?

## Background

In this section, we first introduce the context for this study, then describe key features of DE, and conclude with the theoretical framework.

### Study Context

SB 1720 (2013) was designed to decrease the time students spent on non-credit bearing DE courses before progressing into college-level courses in Florida’s state colleges (formerly community colleges). To this end, the legislation made placement tests and developmental-level coursework optional for active-duty military students and students who entered 9th grade in a Florida public school in 2003/04 or thereafter and earned a standard Florida high school diploma. SB 1720 (2013) also addressed concerns about DE instruction by requiring that FCS institutions offer DE courses through a variety of teaching strategies including compressed, contextualized, co-requisite, or modularized instruction (SB 1720, 2013).

In this study, we use the term “underprepared” to refer to community college students recommended for DE by advisors and instructors in the Florida College System (FCS) whether those students enrolled in DE courses or were among the students eligible to enroll directly in college-level coursework without a placement test. Oftentimes this recommendation was made based on scores on the assessment known as the Postsecondary Education Readiness Test, which was widely used in Florida prior to the legislation. After the legislation, students were deemed underprepared based on multiple measures of documented student achievement including high school GPA, the highest level of math completed, and available standardized test scores (Hu et al., 2017). Considering the amount of student choice provided by SB 1720, we apply this label whether students ultimately enrolled in DE courses or not.

Professional development following the legislation was made widely available to both DE and college-level faculty system-wide. Beyond college credentials, most FCS institutions did not mandate special training tailored to meeting the needs of underprepared students for either DE or college-level instructors; however, at the system level, the Florida Student Success Center organized numerous events and convenings to share best practices with FCS faculty (Hu et al., 2017). Professional development on promising practices for working with underprepared students was also prevalent at the institution level. Indeed, a few institutions in the FCS required that faculty complete in-house professional development credits to qualify for and maintain full-time instructor status (Hu et al., 2015).

Following the legislation, many college-level instructors adjusted their teaching strategies with the influx of underprepared students in their classes. This included using in-class assessments of basic skills during the first week of class for early identification of students in need of extra help, adjusting the pace of instruction, creating basic skills tutorials that could be completed outside of class, and connecting underprepared students with academic support services (Hu et al., 2017).

After the legislation, passing rates in first-level credit-bearing mathematics courses declined. However, based on estimates of the whole cohort, the overall proportion of students passing gateway courses increased compared to before the legislation (Hu et al., 2016). Students who were nearly college ready were more likely to pass first-level credit bearing courses than were the most severely underprepared students (Hu et al., 2016).

### Literature Review

In this literature review, we first explore DE, then consider developmental advising, and academic scaffolding, and conclude with campus collaboration.

DE programs are thought of by many solely as remedial math and English courses to be completed prior to entering college-level coursework. However, equating DE to remedial coursework is a serious misconception as DE curriculum is a holistic approach to education taken in support of underserved students (Boylan, 2002; Goudas & Boylan, 2012). This approach extends beyond remedial coursework to include a variety of in- and out-of-class support services (Boylan, 2002; Goudas & Boylan, 2012), often provided by learning-assistance centers: tutoring, specialized learning workshops about such topics as time management or study skills, and other supports (Boylan, 2002). DE services are widely used by students enrolled at two-year institutions, particularly by those who are female, non-White, economically disadvantaged, first-generation college attendees or adults and returning students (Chen & Simone, 2016).

Though distinct from DE, developmental advising as an advising philosophy has been recommended as a promising practice for underprepared students (Bailey, Jaggars, & Jenkins, 2015), and can be beneficial for the aforementioned students. Developmental advising is considered a developmental process where students and advisors share control of the process while students are encouraged to make their own educational decisions. Developmental advising contrasts with prescriptive advising, whereby the advisor controls the outcome of the advising session and provides recommendations to students as an expert on the curriculum (Grites, 2013).

Academic scaffolding, defined by Mercer (1995) as the “provision of guidance and support which is increased or withdrawn in response to the developing competence of the learner” (p. 75), is essential for underprepared students enrolled in both DE and college-level coursework. Ideally, faculty support is slowly scaled back as the sequence progresses and individual students become more comfortable with the subject matter at hand. Instructors also incorporate individual scaffolding practices and activities in designing learning. Students underprepared for college-level work need this intentional scaffolding, especially in mathematics (Bailey, 2009).

Coordination and collaboration among campus personnel through sharing instructional strategies, aligning goals for students, and avoiding redundancy are all key to success for underprepared students (Boylan, 2002; Goudas & Boylan, 2012). Considering the college more broadly, collaboration between academic units provides a forum for dissemination of promising practices to a wider audience, allowing underprepared students to benefit.

Even though it has documented value, collaboration within institutions of higher education is difficult to attain (Kanter, 1994; Kezar, 2005). Kezar (2005) identifies three steps (i.e., building commitment, commitment, and sustaining commitment) that must be undertaken by colleges to generate an environment conducive to collaboration. This “collaborative advantage,” a term coined by Kanter (1994), depends heavily on well-managed relationships. Campus collaboration was not without challenges following SB1720, however, because staff members in different roles oftentimes held different perspectives and had different overriding interests in terms of the larger organization (Hu et al., 2015).

### Theoretical Framework

Researchers have identified college readiness domains that help students who enter higher education underprepared for college-level work succeed in college. College readiness domains are “higher order factors associated with college success,” which can be targeted by instructors and other campus personnel as “key areas for developmental intervention to reduce both the academic and the persistence ‘risk’ of entering students” (Le, Casillas, Robbins, & Langley, 2005, p. 483). Our study is grounded in the concept of college readiness domains identified by Robbins and colleagues (2004) which include motivation, academic-related skills, and social engagement.

For students underprepared for college-level work, the journey to an associate’s or bachelor’s degree will likely take longer than for other students, requiring higher levels of motivation and persistence (Bailey et al., 2010). Instructors who work with students to set goals find their students are more likely to reach said goals and exhibit increased confidence and self-efficacy as a result (Cho & Shen, 2013). There is an extensive literature on the role of motivation in learning; however, broadly speaking, we refer here to self-regulatory skills that allow students to “motivate themselves to work toward achieving learning goals” (Robbins, Oh, Le, & Button, 2009, p.1164).

Academic-related skills are a second, and critical, component of college readiness. Academic-related skills are those that help students to complete specific learning tasks in higher education (Allen, Robbins, Casillas, & Oh, 2008; Cho & Shen, 2013; Hattie, Biggs, & Purdie, 1996). Robbins et al. (2009) defines these skills as “geared toward improving skills and knowledge deemed to be critical for successful academic performance” (p. 1165). In this study, we adopt Snowman’s (1984) categorization of specific skills taught explicitly by instructors as “learning tactics” and several tactics grouped together as “learning strategies” (see Tables 1 and 2 below).

**Table 1:** Scaffolding academic-related skills in college-level instruction

Teaching strategies	Specific tactics
Communicating academic and behavioral norms	Spending the first few days of class discussing academic skills, how to come to class prepared, academic and behavioral expectations such as communication norms in community college
Assessing student academic preparation early	Administering basic skills diagnostics on the first day of class
Teaching basic skills “on the side”	Creating online basic skills materials for students to use out of class as well as in the learning lab
Providing lots of repetition	Repeating content by presenting the same material in more than one way
Giving frequent feedback on student work	Giving students frequent opportunities to revise and resubmit work for higher grades
Teaching basic writing	Providing opportunities for prewriting and journaling while clearly delineating steps in the writing process, and teaching citation methods
Fostering technology literacy	Requiring students to perform basic technology skills in class such as sending an email or creating a Power Point

**Table 2:** Scaffolding social engagement and motivation in college-level instruction

Teaching strategies	Specific tactics
Providing lots of individualized attention	Showing concern for student wellbeing by asking individual students about their lives to learn their backgrounds and life circumstances
	Scheduling individualized conferences to build rapport and assess gaps in student understanding
	Scheduling work time during class for instructor to rotate around class giving individual attention
	Allowing for flexibility and adaptability to students needs and life circumstances
Building academic confidence	Tempering evaluative feedback with comments about what students have done well
	Providing clear and specific feedback on how to improve work
	Leaving room for mistakes by explaining the essential role of mistakes in learning
	Instructors sharing their own mistakes to model academic success despite life challenges
	Dropping the lowest grade
Encouraging student questions	Creating a classroom environment that welcomes student questions
	Slowing down the class (within reason) to confirm understanding among all students
	Coming to class early and staying late to engage in informal conversation and address questions for students less comfortable seeking help in front of classmates
	Providing multiple means for faculty-student communication, including in-person meetings, teleconference by Zoom or Skype, email, phone, or text

Social engagement is the final domain of college readiness identified by Robbins (2004). Social engagement refers to students’ participation in teamwork, feelings of social connection on campus, and sociability with campus personnel and peers (Allen et al., 2008; Cho & Shen, 2013; Le et al., 2005). Interventions intended to increase social engagement in students include widely-used college programs such as new student orientation and first-year experience courses whose aim is to socialize students to the college environment and college student role while encouraging a greater sense of belonging at their institutions (Allen et al., 2008; Cho & Shen, 2013; Robbins et al., 2004).

Taken together, scaffolding, developmental advising, and campus collaboration are some of the means through which faculty and staff in the FCS fostered the college readiness domains of motivation, academic-related skills, and social engagement. We next consider the research methods employed in this study.

## Method

In this qualitative study, we adopt a holistic research design to explore the global nature of a phenomenon bounded by a particular context (Yin, 2017). Specifically, our study examines the philosophy of working with students underprepared for college-level work in the context of DE reform in the FCS. Our analysis process throughout employed the constant comparative method, which “involves systematically examining and refining the variations in emergent and grounded concepts” (Patton, 2015, p. 289).

## Data Collection

During the four academic years between fall 2014 and spring 2018, we alternated between data collection and data analysis each year. In total, we conducted 36 one- and two-day site visits to 21 FCS institutions. The 21 FCS institutions were comprised of both community colleges offering only associate degrees and state colleges offering a combination of associate and one or more bachelor’s degrees. The data sources for this study include institutional documents, field observations, and transcripts from focus groups with relevant stakeholders at the 21 colleges collected during site visits. Site visits were conducted by teams of two to four researchers travelling to each institution with some repeat visits to institutions. Data were then analyzed each year.

We sampled at both the institution-level and individual, participant-level. At both levels, our qualitative sampling strategy was a “purposive, maximum variation sample,” (Patton, 2015, p. 243) which involved sampling widely to purposely pick a wide range of examples to achieve “variation on dimensions of interest” (Patton, 2015, p. 243). The 21 institutions in our sample represented three quarters of the 28 institutions in the FCS. The 28 FCS institutions were divided into three tiers based on quantitative student outcome data that were most directly related to the legislation (an average of passing rates in DE English and math, and passing rates in first-level credit-bearing courses in English and math). These quantitative data were used only for sampling at the institution-level and did not constitute a data source for this study. From these tiered data, we then invited institutions from each tier to participate in our research, ensuring that we included institutions in every region of the state and institutions that varied in terms of location (i.e., rural, suburban, and urban) and enrollment size.

Administrators at participating institutions solicited focus group participants and secured on-campus space for the focus group sessions. They were requested to select staff who had been involved in implementation of the legislation and students who had been deemed underprepared by campus personnel at the institution. Administrators were also requested to select demographically diverse students, including adult students, students of color, English language learners, economically disadvantaged students, and veterans. While administrators were in the best position to recruit participants due to their depth of knowledge and connections at their home institutions, we acknowledge that this presents a limitation in the sample due to the tendency of administrators to self-select participants who would present their institutions in a favorable light.

## Institutional Documents

Before the site visits, the research team collected and analyzed implementation plans from all 21 FCS institutions. The plans were used to provide essential context for understanding the broad institutional changes that took place following SB 1720 and to support the development of the focus group interview protocol and the initial coding framework. Additional documents, such as advising flowcharts and course syllabi, were collected during the site visits to provide background information for our analyses; these helped us to interpret conversations during advising sessions as well as the course content of the math and English classes we observed.

## Field Observations

Researchers generated field notes identifying salient, interesting, or illuminating observations from each visit. Field notes were particularly valuable in allowing the researchers to directly observe interactions between students and campus personnel during seven, hour-long advising sessions and nine one to two hour-long classroom observations of math and English DE and first-level credit-bearing classes. Field observations related to how campus personnel presented academic-related skills and observable signs of students’ motivation and engagement were particularly relevant to this study.

## Focus Groups and Individual Interviews

Focus group and interview data in this study included transcripts from 19 individual interviews and 179 focus groups. In total, we spoke with 378 students, 239 implementation leaders (primarily academic administrators), 279 faculty, 217 advisors, and 23 support staff (1136 total participants).

The focus group protocols included a broad array of questions, which changed over the four-year period. Questions from the campus personnel focus group protocol relevant to the present study included: “*Tell me about the courses you currently teach. How has your curriculum evolved over time? What changes have you made to your [classroom/advising/support/administrative] practices to address student academic preparation since SB 1720 passed in 2013?” and “How do you collaborate with others on campus to address student needs?”*

Questions from the student focus group protocol relevant to the present study included: “*Can you tell us about a time that you have struggled in your coursework?”* and “*Tell us about the professor, advisor, or other staff member who has made the biggest impression on you (either a good impression or a bad impression). What did that person say or do to make an impression on you?*”

## Data Analysis

First, a digital recording of all focus groups was used to generate verbatim transcripts, which were then imported into qualitative data analysis software, NVivo 10, for coding and analysis. Over the four-year period, we developed an evolving coding framework, which incorporated a combination of a priori and emergent codes. We included broad codes (or parent codes) in our framework like *curriculum* and *students* as well as more detailed codes (child and grandchild codes) such as *instructional quality* and *student motivation.*

The coding framework was re-evaluated each year. In this annual process, infrequently used grandchild codes were collapsed into parent codes while large parent codes were subdivided into multiple grandchild codes. To ensure consistency of coding among multiple coders, we kept a codebook with definitions of codes and met weekly to discuss and clarify codes on which we had lower levels of coding agreement in NVivo.

Our data analysis procedures for this qualitative study consisted of five steps. First, we open coded (or first-cycle coded) the data for the larger research project. We then identified significant statements from the data relevant to the current study on pedagogy. In second cycle coding, themes identified in open coding were reorganized and collapsed into the four broad themes we illuminate in the current study: the philosophy of education or pedagogy, characteristics of instructors, instructional strategies, and collaboration. Finally, after second cycle coding was complete, we created textural descriptions, which summarized the data for each of the four themes (Miles, Huberman, & Saldaña, 2014; Saldaña, 2015; Yin, 2017). Researchers also wrote analytic memos throughout the research process in all 4 years, assisting us in developing the initial coding framework, identifying the broad themes in our data related to pedagogy, and creating textural descriptions of each theme (Corbin & Strauss, 2015).

The trustworthiness of our qualitative interpretations was established through an inter-coder reliability process, data source triangulation (i.e., institutional documents, observations, focus groups, and individual interviews), analyst triangulation (i.e., eight researchers coded the data), member-checking, and peer debriefing with two researchers who acted as “devil’s advocates” in questioning the study’s interpretations (Patton, 2015).

## Findings

In this section, we first present a formal definition of the overarching philosophy of college readiness strategies, which we label a pedagogy of preparation. We also define the connection between the overarching philosophy of a pedagogy of preparation and the open access mission of community colleges and their social justice imperative to educate all students. Second, we identify common strategies and specific tactics for the core college readiness practices of scaffolding of academic-related skills, motivating students, creating social engagement, and encouraging campus-wide collaboration (listed in Tables 1, 2, and 3).

**Table 3:** Supporting college readiness by improving campus-wide coordination

Campus personnel coordination	Coordination strategies
Administrators, faculty and advisors	Enacting policies so that students enroll in courses with high failure rates (e.g., math courses) early to prevent delayed graduation
	Enacting policies so that sequences of math courses are taken close together enabling students to better retain basic skills
	Sharing data trends such as high-failure classes or times in the semester when students withdraw so that faculty can make teaching adjustments
Faculty and faculty	Working together to create standardized curriculum and grading rubrics in entry-level college courses so that students are similarly prepared for the next course in a sequence
	Coordinating on topics presented at different levels for continuity across course sequences
	Coordinating across academic disciplines so that English and math courses incorporate authentic learning tasks students will use in their majors
	Creating professional development workshops for faculty across disciplines related to strategies for working with students with disabilities, English language learners, and presenting basic academic-related skills to students underprepared for college-level work, facilitated by faculty experts in these areas
Faculty and advisors	Faculty presenting basic skills diagnostics in new student orientation or advising sessions for early identification of students in need of academic support services
	Consistent use of and communication between faculty and advisors through the early alert system
	Faculty presenting detailed information about curriculum to advisors to improve advising recommendations
	Advisors inviting faculty to describe curriculum and academic expectations to students at new student orientation
Faculty and academic support staff	Leveraging embedded tutors or learning assistants within classrooms to aid the curriculum through academic support practices
	Faculty holding office hours in learning labs or academic support centers

### A Pedagogy of Preparation: “We Are Here for Everybody”

We define a pedagogy of preparation as a philosophy that holds that the purpose of a community college education is to meet students where they are academically, regardless of prior preparation and schooling experiences. In the community college context, this pedagogy involves helping students increase their academic-related skills, their motivation for learning, and their social engagement through instruction, advising, and support services.

FCS instructors suggested that addressing college readiness domains is closely aligned with the open access mission of community colleges because it lays the foundation for success in higher education for students who come to college underprepared for college-level work. One instructor contrasted the “sink or swim” academic philosophy of the four-year institution she attended as an undergraduate and the college where she now teaches in this way:

I actually heard one professor [in college] stand up and say, ‘Teach the best and shoot the rest. We’re not here for everybody.’ But you see, we are here for everybody, and that’s the difference in the culture [in community colleges].

Rooted in the social justice perspective that all students are entitled to postsecondary educational opportunity, the “we’re here for everybody” philosophy is central to the pedagogy of preparation.

After SB 1720 passed, FCS campus personnel committed themselves, more than ever, to a pedagogy of preparation as instructors, advisors, and other support staff found new ways to foster stronger academic-related skills, motivation, and social engagement across campus in the absence of DE courses. Throughout the focus group data for this study, campus personnel in a variety of professional roles shared a consistency of language that emphasized the key practices of college readiness. Phrases including “scaffolding,” “motivation,” “engagement,” and “collaboration” were frequently used by academic administrators, faculty members, advisors, and support staff alike.

### Scaffolding Academic-Related Skills: “Teaching Them How to Become Students”

Since many students now choose to bypass DE, they no longer receive academic-related skills in preparatory classes. However, in the words of one faculty member, “The students haven’t changed; they’re just in different classes.” As such, first-year experience (FYE) and student life skills (SLS) courses have become increasingly important since 2014. Additionally, focus group participants shared that existing first-level, credit-bearing (or gateway) courses and extended student orientations have been augmented with college readiness skills. Table 1, which was derived from data provided by instructors, includes a variety of general strategies for scaffolding academic-related skills.

Each of these strategies shares a commitment to careful scaffolding of academic-related skills via instruction. As an administrator noted, “If the challenge increases, you must provide an equal and proportional amount of support.” Indeed, a faculty member stated that with respect to students bypassing DE, “They’re missing the frying pan, going straight to the fire, so to speak. But, by scaffolding it, giving them this level, and teaching them how to become a student, then they can be more successful.”

Overwhelmingly, faculty agreed that a core function of community college instruction is to teach students new to higher education what it means to be a college student in terms of academic expectations. A faculty member explained:

…[O]n my very first day of class, it wasn’t even about the syllabus. It wasn’t about grades; it wasn’t about any of this. It was about, Okay, you are away from home. Let’s talk about being in college. Let’s talk about being away from the mother, father. Let’s talk about getting up in the morning, going to class, getting to class on time, not only that, coming to class prepared, not just coming to class with a cup and keys and saying, ‘I’m in college.’

He continued by describing the importance of these first few days of class in laying the academic foundation for the rest of the semester:

And I would spend probably just those first two or three days in class just having that discussion about what it means to be in college. After we’ve gotten that down, then I would actually get into the content, but until you have that conversation or unless you have an instructor who’s willing to have that conversation with students, sometimes all is lost.

Table 1 also notes that college-level instructors scaffold academic-related skills by fostering technology literacy in their classrooms. To this point, during a classroom field observation of a first-level English class [ENC 1101], students were working in groups preparing PowerPoint presentations for later in the semester. The energetic young instructor circulated around the room, helping different groups when they got stuck on parts of the assignment or raised questions. Approximately every 15 min during the two-hour class the instructor checked compliance on technology-related tasks she assigned the class. Some of these checks included individual students opening up PowerPoint presentations, changing the font sizes and limiting the number of words on each slide, embedding a YouTube video into the presentation, emailing a current draft of the group PowerPoint presentation to the instructor in real time, and uploading the final product to Blackboard, the course management system.

When asked about this assignment and its relevance to learning outcomes in college-level English, the instructor explained that she incorporated technological literacy skills within ENC 1101 because it is crucial to success in college, particularly to students’ ability to submit their work to instructors.

Similarly, a faculty member in a focus group described an early in-class assignment designed to ensure that students can use their campus email accounts:

We had a simple etiquette email assignment, if they needed to email a professor, and we talked to them about how to speak properly, how not to use text language…. Teaching that was a trip… showing them how to access their account email, even showing them how to sync it to their phone, this and that, giving them these how-to links, how to get it.

While academic-related skills are often the centerpiece of preparing students for college-level coursework, increasing student motivation and social engagement with campus personnel and peers are also essential to college readiness among students.

### Scaffolding Motivation and Social Engagement: “Getting a Good Foundation”

In this section, we present student views on instructors who motivated them to learn and promoted social engagement in the classroom, as well as campus personnel perspectives on specific tactics for promoting these attributes in students. We combine these two aspects of “college readiness” into one section because many of these strategies advance motivation and social engagement simultaneously, and it is therefore difficult to disentangle them. In Table 2, we present strategies for scaffolding motivation and social engagement, which were derived from our data.

Students reported several instructor characteristics that fostered social engagement in college and their motivation to learn. A positive instructor practice frequently cited by students involved not penalizing students academically when life circumstances intervened. One student described how his instructor demonstrated genuine concern by gently confronting him about the need to take responsibility for his learning while being flexible enough to give him a second chance to complete course requirements. The student explained how the professor’s accommodations contributed to his success:

I fell behind… Something happened in my personal life that… put me down completely with school, and I just stopped showing up to his class…. [The instructor] finally sent me a text message… and he just basically, overall, he’s saying, ‘When are you going to take responsibility for yourself, and just start showing up?’ And I said, ‘What do you mean?’ He said, ‘Well, I know what happened because [of your] friends in the class. I know what’s going on. You just need to cut that crap and come to class.’ So, I start showing up in class, and he gave me all like extra time to complete the project and stuff…. Instead of a D, I have a B in this class. And I’m probably going to end with an A.

For this student, an instructor who gave him individualized attention and showed genuine interest in his welfare and personal challenges made a significant difference both to his motivation to attend class and ultimately his grades. Flexibility and adaptability to student needs was among the instructor characteristics most valued by students. Like this student, many students in our focus groups also appreciated instructors who built relationships with them through actions they perceived as nurturing and supportive.

Though positive assessments of faculty were more prevalent in the data, students also described faculty who were less accommodating. Often this involved faculty whom students perceived were rigid about course expectations or failed to give individualized attention by exerting minimal effort on students’ behalf. Students were critical of these faculty and interpreted their behavior as lacking commitment to their success.

Creating tasks with increasing levels of challenge also helped to foster a sense of academic confidence. One faculty member remarked that college readiness is about “filling those gaps, but it’s making them believe that they can do it.” Similarly, another instructor stated:

They’re getting a good foundation. They’re going to keep going on if they are – they’re feeling comfortable. It’s like, ‘Oh, I knocked this out. Let’s go.’

Another student explained how her college-level professor’s “energetic” teaching style and words of encouragement increased her academic confidence, motivation to learn, and classroom engagement:

We love her. Every student wants to be in her class. She makes sure that everybody knows that it’s important. And she will tell you all the time, ‘You are wonderful.’ And you cannot wait to go to that class to show that you do care. And you took her advice, and you try your best and look, your paper turned to be one of the best of it. So, in my words that’s what a good professor is.

Students also appreciated an engaging personality in the classroom among faculty they perceived as vivacious or humorous. An example of this occurred during an observation of a math course. A young professor dressed in blue jeans, a white T-shirt, and flip flops taught a class of racially diverse students of varying ages. Throughout the observation, visible signs of engagement from students who were academically on-task throughout the class suggested that the instructor instilled motivation in the students and increased their mathematical self-efficacy. The atmosphere in the class was one of strong rapport both between the instructor and the students and among the students.

The instructor cultivated a supportive classroom culture that included gentle humor. At the beginning of class, for instance, when a smiling student told the professor that he finished his homework, she laughed and joked that she thought he might be fibbing to her. She also showed personal concern by asking a student why his friend was not in class. He explained that his friend was moving to another state.

During the class period, the instructor’s technique was to first work a problem on the board by herself, next work half of a problem, leaving students to complete the problem in groups, and finally to put a problem on the board for students to work from start to finish until she revealed the answer. During this process, she exhorted the students to “believe in themselves” because she “believes in them.” While working a problem, the professor smiled and pointed to a student with a confused expression and told him his face told her that he “didn’t get it.” She then told him that she was going to keep explaining the problem until his face showed her that he understood. One student sitting near the back of class blurted out answers that weren’t always correct, but his enthusiasm, coupled with the instructor’s enthusiasm, was infectious. The instructor’s encouraging reaction to his incorrect answers communicated to his classmates that it was OK to take risks in the classroom participating in class even with incorrect answers.

In addition to building academic confidence, faculty noted that an integral part of fostering success in college is encouraging students to take greater responsibility for their educational decisions. To this point, a faculty member stated:

It [DE] helped them to transition into the college life where you take more responsibility for your own learning. Where you are responsible for finding out what are the questions that you need to ask, contact the professor and things like that.

The faculty member explained to students that an important part of taking responsibility for one’s own learning was to demonstrate the agency and initiative to ask questions and get help from instructors when needed.

While most students in our focus groups described instructors who welcomed questions, a few identified instructors who showed visible impatience with students of varying levels of academic preparation. One student described an instructor, noting, “So in a way I felt like she was calling us kind of stupid if we didn’t know the things.” Another student said of an instructor:

They were mean to people, talking down to them. I did not appreciate that at all. You’re here to teach me. And I thought, if I ask you a question or anybody asks you a question, don’t be rolling your eyes at them.

Some students, like this one, cited faculty they found to be rude or impatient with students who were underprepared for college-level work. Students in our focus groups shared that these behaviors undermined their sense of social engagement and motivation.

In addition to scaffolding instruction through academic-related skills, social engagement, and motivation, faculty and other campus personnel shared new ways staff have reached across academic silos to work collaboratively to improve outcomes for students who are underprepared.

### Campus-Wide Coordination: “A Good, Positive Consequence”

After SB 1720, institutions across the FCS saw an increased demand for campus services such as advising and academic support, a trend which has encouraged greater institutional coordination among faculty, administrators, advisors, and support staff. With respect to the legislation, one administrator stated:

One of the greatest things that has come out of this [SB 1720] is collaboration. We were able to work together, college-wide, as mentioned, but also within the academic success centers, we were able to make sure that we were all aware of what services are offered on each campus.

Because college readiness efforts were no longer focused within DE departments after SB 1720, campus-coordination increased in the overlapping functional areas that traditionally served the needs of students underprepared for college-level work. While classroom instruction remained the centerpiece of college readiness efforts, some of the areas of overlap included new student orientation, learning labs and centers, early alert, and electronic referral systems.

In several field observations collected during advising sessions, advisors demonstrated their institutions’ technology-supported advising systems. The advising systems used in much of the FCS combine the benefits of e-advising with a relationship-focused developmental advising philosophy. In some of these systems, course pathways and transfer requirements for four-year institutions across the state of Florida have been programmed into the system. These features facilitate the advising process by reducing information overload during the advising conversation between students and advisors. Rather than devote precious advising sessions to explaining complicated advising handouts, advisors at institutions with automated systems can engage in developmental advising by having conversations that are less technical, and more focused on topics such as students’ long-term life goals, and the specific actions and commitment required of them to achieve these goals.

Perhaps more importantly, these advising systems foster collaboration across campus to support student development and success. Another useful feature of many of these systems are the capability for campus personnel in several job roles (e.g., advisor, instructor, support staff, and administrator) to log in and make notations in students’ records so that others on campus know the history of academic and personal challenges a student has reported to staff. This system allows staff across campus to communicate and coordinate to connect students with campus resources that will contribute to their success. Aside from the way the advising system facilitated developmental conversations between advisors and students, the system was also effective at increasing levels of coordination among staff across campus.

Enhanced advising systems were but one example of ways that collaboration increased after the passage of the legislation. An administrator in a focus group agreed, stating:

I think one of the good consequences [of SB 1720] is… collaboration among the whole campus… Academics works with student services, which works with access, which works with testing… The collaboration on the campuses has been a good positive consequence of this.

Oftentimes this meant that instructors, advisors, and academic support staff became more flexible about where services were offered to students underprepared for college-level work. In bridging these functional areas, embedded tutors attended classes, some faculty began to hold office hours in learning labs, and faculty and academic support staff began to participate in new student orientation sessions. Table 3 describes some of the coordination strategies from our campus personnel focus group data.

While these areas of cross-campus coordination had already begun on some campuses prior to SB 1720, participants shared that the legislation accelerated these collaborative efforts.

While most of our data suggest that cross-campus coordination had increased after SB 1720, collaboration was not without challenges. One such example was with the early alert system at a campus where faculty members and advisors struggled to coordinate their efforts on behalf of students. An advisor explained:

They'll [the faculty] say, ‘Contact the student because he's failing.’ I contact the student. I say, ‘Hey, you need to talk to your instructor, man. You're not doing well in class.’ He [the student] calls me back and goes, ‘Well, the instructor said he's not gonna take any of that work late and I have an F and there's nothing I can do about it.’ Well, then why did I reach out to this student and tell him he needs to talk to the instructor? That's ridiculous.

While challenges to collaboration were less prevalent in our data, they tended to occur when staff members in different roles held different perspectives or had different overriding interests in terms of the larger organization.

Having explored the ways a pedagogy of preparation was expressed in FCS institutions after SB 1720, we next consider the implications of changes in the FCS for community colleges nationally.

## Discussion

Previous research suggests not only that DE is costly for institutions and individuals (Scott-Clayton et al., 2014) but also that students enrolled in DE have low levels of success. Additionally, DE has been shown to unfairly disadvantage Black, Latinx, and low-income students due to low levels of eventual degree completion and transfer rates (Attewell et al., 2006; Crisp & Delgado, 2014; Melguizo et al., 2011). Because educational attainment is inextricably linked with the open access mission and social justice imperative of community colleges, reforms such as Florida’s SB 1720 that allow underprepared students to enter directly into college-level coursework while buttressing college readiness skills have proven to be an effective alternative to required DE coursework (Bailey et al., 2015).

With respect to the quality of instruction for students underprepared for college-level work, Jaggars and Bickerstaff (2018) have stated:

…the quality of evidence on curricular and pedagogical reform in developmental education remains limited, as such reforms often accompany structural changes but are rarely the explicit focus of implementation or evaluation. Accordingly, researchers need to focus more strongly on investigating instructional approaches that work well, as well as documenting faculty professional development models that support the success of these approaches (p. 496).

Thus, as DE reform efforts accelerate around the country (Jaggars & Bickerstaff, 2018), it is essential that we consider how best to tailor college-level curriculum and academic supports to meet the needs of students who are underprepared. Following SB 1720, the traditional role of instructors extended beyond teaching basic content-area skills to include academic-related skills such as organization, study skills, help seeking, and time management. Equally important has been fostering students’ motivation to succeed in college-level coursework and social engagement with others.

Our findings on a pedagogy of preparation support the college readiness domains of academic-related skills, motivation, and social engagement proposed by Robbins and colleagues (2004). We extend these findings by identifying specific practices that educators can use to foster these skills (outlined in Tables 1, 2, and 3). In addition to the strategies provided in Tables 1, 2, and 3, we recommend the creation of professional development trainings focused on the needs of students underprepared for college-level work in and out of the classroom. Such trainings would bring together faculty and staff from across campus to better understand the backgrounds of students and to brainstorm new opportunities for institutional collaboration to increase student success.

One unexpected, yet positive consequence of the legislation in Florida has been increased coordination between campus personnel. We see examples of this collaboration in early alert and orientation programs, among others. Fostering student success has long been an essential task for faculty, advisors, and support staff alike. Therefore, we recommend that colleges designate an organizing structure for college readiness (e.g., a person or office, plus a cross-unit committee or task force that meets regularly) so that clear responsibility for fostering readiness is defined. This structure can also facilitate the cross-campus coordination and collaboration required for a pedagogy of preparation to be effective.

Building on these findings, we think it important to extend the notion of collaboration beyond the confines of one institution to include collaboration between: (1) colleges, (2) colleges and the universities to which their students transfer, and (3) colleges and their feeder high schools (Boylan, 2002). Although this topic is beyond the scope of the current paper, it provides a fruitful line of inquiry moving forward. A deep dive into specific advising and academic support practices that support college readiness should also be explored in future research.

Considering how integral college readiness skills are to the community college mission of providing access to higher education to underprepared and underserved populations, these skills must remain central, rather than peripheral, to the job descriptions of all faculty and staff working on campus.

## Data Availability

Data aren’t currently available. They are in the process of being redacted.

## References

[CR1] Allen J, Robbins SB, Casillas A, Oh IS (2008). Third-year college retention and transfer: Effects of academic performance, motivation, and social connectedness. Research in Higher Education.

[CR2] Attewell P, Lavin D, Domina T, Levey T (2006). New evidence on college remediation. Journal of Higher Education.

[CR3] Bailey T (2009). Challenge and opportunity: Rethinking the role and function of developmental education in community college. New Directions for Community Colleges.

[CR4] Bailey T, Jeong D, Cho S (2010). Referral, enrollment, and completion in developmental education sequences in community colleges. Economics of Education Review.

[CR5] Bailey, T. R., Jaggars, S. S., & Jenkins, D. (2015). *Helping underprepared students. Redesigning America’s community colleges: A clearer path to student success*. Boston: Harvard University Press.

[CR6] Bettinger EP, Long BT (2005). Remediation at the community college: Student participation and outcomes. New Directions for Community Colleges.

[CR7] Boylan HR (2002). *What works: Research-based best practices in developmental education*.

[CR8] Chen, X. & Simone, S. (2016, September). *Remedial course taking at U.S. public 2- and 4-year institutions: scope, experiences, and outcomes (NCES 2016-405)*. Washington, DC: U.S. Department of Education, Institute for Education Statistics.

[CR9] Cho MH, Shen D (2013). Self-regulation in online learning. Distance Education.

[CR10] Corbin JM, Strauss AL (2015). *Basics of qualitative research: Techniques and procedures for developing grounded theory*.

[CR11] Crisp G, Delgado C (2014). The impact of developmental education on community college persistence and vertical transfer. Community College Review.

[CR12] Goudas AM, Boylan HR (2012). Addressing flawed research in developmental education. Journal of Developmental Education.

[CR13] Grimes SK, David KC (1999). Underprepared community college students: Implications of attitudinal and experiential differences. Community College Review.

[CR14] Grites TJ (2013). Developmental academic advising: A 40-year context. NACADA Journal.

[CR15] Hattie, J., Biggs, J., & Purdie, N. (1996). Effects of learning skills interventions on student learning: A meta-analysis. *Review of Educational Research*, *66*(2), 99–136.

[CR16] Hu, S., Bertrand Jones, T., Brower, R. L., Park, T., Tandberg, D., Nix, A., Rahming, S., & Martindale, S. (2015). *Learning from the ground up: Developmental education reform at Florida College System institutions*. Tallahassee, FL: Center for Postsecondary Success.

[CR17] Hu, S., Park, T., Woods, C., Richard, K., Tandberg, D. A., & Bertrand Jones, T. (2016). *Probability of success: Evaluation of Florida’s developmental education redesign based on cohorts of first-time-in-college students from 2009-10 to 2014-15*. Tallahassee, FL: Center for Postsecondary Success.

[CR18] Hu, S., Bertrand Jones, T., Brower, R. L., Park, T., Nix, A., Rahming, S., Harrison, J., Sermon, J., & Daniels, N. (2017). *Changes on the ground: Site visit report of the third year of developmental education reform in the Florida College System*. Tallahassee, FL: Center for Postsecondary Success.

[CR19] Jaggars, S. S., & Bickerstaff, S. (2018). Developmental education: the evolution of research and reform. In M. B. Paulsen & L. W. Perna (Eds.), *Higher Education: Handbook of Theory and Research, 34*, 469–503). Cham, Switzerland: Springer.

[CR20] Kanter RM (1994). Collaborative advantage: the art of alliances. Harvard Business Review.

[CR21] Kezar A (2005). Redesigning for collaboration within higher education institutions: An exploration into the developmental process. Research in Higher Education.

[CR22] Le, H., Casillas, A., Robbins, S. B., & Langley, R. (2005). Motivational and skills, social, and self-management predictors of college outcomes: Constructing the Student Readiness Inventory. *Educational and Psychological Measurement*, *65*(3), 482–508.

[CR23] Melguizo T, Bos J, Prather G (2011). Is developmental education helping community college students persist? A critical review of the literature. American Behavioral Scientist.

[CR24] Mercer N (1995). *The guided construction of knowledge: Talk amongst teachers and learners*.

[CR25] Miles MB, Huberman AM, Saldaña J (2014). *Qualitative data analysis: A methods sourcebook*.

[CR26] Patton MQ (2015). *Qualitative evaluation and research methods*.

[CR27] Pretlow, J. III, & Wathington, H. D. (2012). Cost of DE: An update of Breneman and Haarlow.* Journal of Developmental Education, 36*(2), 4–44. Retrieved from https://www.jstor.org/stable/42785092

[CR28] Robbins, S. B., Lauver, K., Le, H., Davis, D., Langley, R., & Carlstrom, A. (2004). Do psychosocial and study skill factors predict college outcomes? A meta-analysis. *Psychological Bulletin*, *130*(2), 261. 10.1037/0033-2909.130.2.261 14979772

[CR29] Robbins SB, Oh IS, Le H, Button C (2009). Intervention effects on college performance and retention as mediated by motivational, emotional, and social control factors: Integrated meta-analytic path analyses. Journal of Applied Psychology.

[CR30] Saldaña J (2015). *The coding manual for qualitative researchers*.

[CR31] Scott-Clayton, J., Crosta, P. M., & Belfield, C. R. (2014). Improving the targeting of treatment: Evidence from college remediation. *Educational Evaluation and Policy Analysis, 36*, 371–393. 10.3102/0162373713517935

[CR32] Senate Bill 1720. (2013). CS for CS for SB 1720, 3rd Engrossed. Retrieved from https://www.flsenate.gov/Session/Bill/2013/1720/BillText/er/PDF

[CR33] Snowman J, Phye GD, Andre T (1984). Learning tactics and strategies. *Cognitive classroom learning: Understanding, thinking and problem solving*.

[CR34] Western Interstate Commission for Higher Education. (2016). Remedial and developmental education. *State Higher Education Policy Database*. Retrieved from http://higheredpolicies.wiche.edu/content/poli

[CR35] Yin RK (2017). Case study research and applications: Design and methods.

